# Development and Evaluation of a Sensitive Bacteriophage-Based MRSA Diagnostic Screen

**DOI:** 10.3390/v12060631

**Published:** 2020-06-11

**Authors:** Matthew Brown, Wendy Hahn, Bryant Bailey, Alex Hall, Gema Rodriguez, Henriett Zahn, Marcia Eisenberg, Stephen Erickson

**Affiliations:** 1 Laboratory Corporation of America Holdings, Burlington, NC 27215, USA; browm49@labcorp.com (M.B.); Bryant.bailey91@gmail.com (B.B.); halla33@labcorp.com (A.H.); rodrg10@labcorp.com (G.R.); Eisenbm@Labcorp.com (M.E.); 2 Laboratory Corporation of America Holdings, New Brighton, MN 55112, USA; Hahnw@labcorp.com (W.H.); Zahnh@Labcorp.com (H.Z.)

**Keywords:** phage-based detection, *Staphylococcus aureus*, diagnostic screen, nasal swab, luciferase reporter phage, MRSA, bacteriophage

## Abstract

Engineered luciferase reporter bacteriophages provide specific, sensitive, rapid and low-cost detection of target bacteria and address growing diagnostic needs in multiple industries. Detection of methicillin-resistant *Staphylococcus aureus* (MRSA) nasal colonization and antibiotic susceptibility play a critical supportive role in preventing hospital-acquired infections and facilitating antibiotic stewardship. We describe the development and evaluation of a novel phage-based MRSA diagnostic screen for nasal swab specimens. The screen utilizes two luciferase reporter phages capable of recognizing genetically-diverse *Staphylococcus aureus*. The beta-lactam antibiotic cefoxitin is included to differentiate between resistant (MRSA) and susceptible organisms. The screen positively identified 97.7% of 390 clinical MRSA isolates at low bacterial concentrations. At higher inoculums, 93.5% of 123 clinical non-MRSA *Staphylococcus aureus* yielded appropriate negative results. Although cross-reactivity of the phage cocktail was observed with other staphylococcal and bacillus species, these false positives were absent under selective conditions. MRSA remained detectable in the presence of 38 distinct competing species and was accurately identified in 100% of 40 spiked nasal specimens. Thus, this six-hour screen sensitively detected MRSA both in vitro and in human nasal matrix.

## 1. Introduction

A recombinant luciferase reporter bacteriophage was utilized to detect target bacteria over 30 years ago [1]. In this study, engineered bacteriophage encoding the bacterial luciferase gene *lux* were used to detect the presence of enteric bacteria. Since the production of viral proteins requires intact host machinery, Lux will only be produced if the recombinant bacteriophages infect viable bacteria in a test sample. Using this approach, luciferase reporter phage assays have been developed for a large variety of applications including food safety, detection of pathogens in clinical samples and profiling of antibiotic susceptibility [2,3,4].

The recently developed and commercialized novel engineered luciferase, NanoLuc, is a small (19 kDa) protein capable of producing a luminescent signal that is 100-fold greater than traditional luciferases [5]. The glow-type kinetics of this luciferase and low background from its novel substrate, furimazine, support the utility of this protein as a reporter. NanoLuc reporter phages have recently been designed and allow for the sensitive detection of target bacteria through robust signal generation [6,7].

Methicillin-resistant *Staphylococcus aureus* (MRSA) is a critically important human pathogen with the capacity to cause various forms of debilitating and fatal infection [8]. MRSA represents a leading cause of surgical site infections in hospitals, associated with longer patient stays, higher rates of readmission, decreased survival rates and an estimated cost of $60,000 per case [9]. As a result of this profound clinical and financial burden to the healthcare industry, significant efforts have been made to understand and control this source of infection. Nasal carriage of MRSA has been found to be a major risk factor for subsequent disease and the majority of *Staphylococcus aureus* infections can be matched to endogenous colonizing strains [10,11,12,13]. Elimination of this risk factor through surveillance and decolonization of MRSA nasal carriers has proven to be a successful strategy in reducing surgical site infections [14].

Currently, MRSA nasal carriage is commonly identified through either polymerase chain reaction (PCR)-based or culture-based methods performed from patient nasal swabs. Detection of MRSA-specific DNA sequences with real-time PCR has demonstrated excellent sensitivity and specificity, rapid time to results (1 to 2 h) and overall clinical effectiveness [15,16,17]. While this method has yielded promising results, two major drawbacks exist. First, new generations of these methods must constantly be developed to match the changing genetic landscape of MRSA resistance [18,19,20]. This effect has resulted in the failure of some assays to detect novel MRSA strains [21,22]. Second, relative to culture-based alternatives, the high cost of this approach has led to uncertainty regarding cost-effectiveness, particularly in regions with low endemic carriage rates [23]. Culture-based methods of detection may involve the use of chromogenic and selective agar and often demonstrate similar strong performance in regard to sensitivity and specificity [24]. While often significantly cheaper than PCR-based methods, one major drawback of culture is that results typically require 18 to 24 h of incubation before they are actionable [23,25,26].

The purpose of this study was to develop and evaluate an in-shift low-cost culture-based diagnostic screen for the detection of MRSA nasal colonization. Two NanoLuc *Staphylococcus aureus* phage reporters were designed as the foundation of this assay. Phages utilized included ISP, a broad-host-range well-characterized phage that is regularly used in phage therapy and MP115, a member of the phage cocktail used in the Food and Drug Administration (FDA)-approved KeyPath MRSA/methicillin-susceptible *Staphylococcus aureus* (MSSA) assay [27,28]. To our knowledge, this study represents the first example of a luciferase phage reporter for MRSA detection. The evaluation of this novel MRSA diagnostic screen has yielded promising results, demonstrating broad inclusivity, sensitivity and effectiveness in both culture and nasal matrix.

## 2. Materials and Methods

### 2.1. Bacterial Strains

Bacterial strains were obtained from the American Type Culture Collection (ATCC, Manassas, VA, USA) with the following exceptions—*Salmonella enterica* S492 was obtained from the University of Georgia Research Foundation and *Staphylococcus aureus* RN4220 was obtained from the University of Iowa. Clinical strains of *Staphylococcus aureus* were internally sourced from clinical microbiology labs (Laboratory Corporation of America Holdings). MRSA isolates from de-identified human clinical specimens originated from three geographically distinct USA sites (Burlington, NC, USA, Phoenix AZ, USA and Raritan NJ, USA). MSSA isolates were obtained in a similar fashion from one site (Burlington, NC, USA). Determination of MRSA or MSSA was confirmed by plating on the selective chromogenic agar, MRSA Select II (Bio-Rad, Marnes-la-Coquette, France). Strains were routinely grown at 37 °C in brain heart infusion (BHI) broth (Becton Dickinson and Company, Sparks, MD, USA) with shaking at 250 revolutions per minute (RPM).

### 2.2. Bacteriophage Source and Stock Preparation

MP115 was obtained from the Colorado School of Mines, while ISP was obtained from Emory University. Stocks of phage were manufactured as follows. For MP115, overnight cultures of RN4220 were diluted, grown to exponential phase and infected at a MOI of 0.01. Cultures were shaken at 250 RPM at 37 °C and monitored for loss of optical density as confirmation of viral propagation. Phage lysates were subsequently clarified by 4 °C centrifugation (type 19 rotor, Beckman) at 14,900× *g* for 10 min. Clarified supernatants were centrifuged again at 4 °C and 14,900× *g* for 2 h. Pellets were resuspended overnight in 1 × TMS (50 mM Tris-HCL pH 7.8, 10 mM MgCl_2_ and 300 mM NaCl). The phage preparation was then treated with 10 µg/mL DNase I and 5 µg/mL RNase. After treatment, the preparation was centrifuged using a swinging bucket rotor at 4696× *g* for 10 min at 4 °C. The supernatant was removed and further purified by cesium chloride density gradient (densities of 1.2, 1.3, 1.4 and 1.6) centrifugation (SW 55 Ti rotor, Beckman) at 109,400× *g* for 2 h at 20 °C. The band containing phage was removed and the preparation placed in dialysis tubing (Spectra/Por 4, MWCO 12,000 to 14,000). Dialysis was performed in TMS with 2.4 M NaCl for 1 h, repeated in TMS with 0.9 M NaCl and repeated again in TMS with 0.3 M NaCl. For ISP, a similar procedure was used with the following exceptions—strain 12600 was used as host, exponential cultures were infected at an MOI of 0.05 and an additional centrifugation using a swinging bucket rotor at 4696× *g* for 10 min at 4 °C was performed after overnight pellet resuspension, prior to treatment with DNase and RNase. Stock titers were determined by standard methods using plaque counting performed on host strains grown in semi-solid agar. Using this method, approximately 3 mL of high titer stocks (ranging from 1 × 10^10^ to 1 × 10^11^ plaque forming units (PFU) were regularly obtained for all bacteriophage and recombinants in this study.

### 2.3. Engineering of Luciferase Reporter Phage Recombinants

Homologous recombination was used to insert NanoLuc into the ISP and MP115 genome. The region preceding the predicted major capsid protein was selected as the target insertion site. In order to avoid disruption of the native promoter, the chosen site was approximately 100 bp upstream of the start codon. Luciferase reporters in *Listeria* have successfully been generated using a similar region, albeit downstream of this gene [29]. To create the donor plasmid for recombination, two homology arms, a promoter and NanoLuc were inserted into the PstI site of the shuttle vector pBAV1KT5gfp (accession HQ191434). A host-specific promoter (5’-TTGTTATAAATAAGTAGTTTGATTAAATACCTTGAATTGCCCCGGCCTAAAAGGAGGTATATTA-3’) was designed based upon previous studies and used to drive the expression of a codon-optimized NanoLuc [27]. Codon optimization for *Staphylococcus aureus* was performed by Genewiz (South Plainfield, NJ, USA) using the available NanoLuc sequence [5]. Homology arms consisted of roughly 500 bp of sequence upstream and downstream of the insertion site. Plasmid synthesis and verification were performed by Genewiz and the resulting donor plasmid referred to as pBAV-MRSA-NL. pBAV-MRSA-NL was utilized for both ISP and MP115 engineering, as the regions of homology share 99.9% identity.

Electroporation-competent *Staphylococcus aureus* were made from RN4220. To achieve this, overnight cultures of RN4220 were diluted and grown to mid-log phase in Tryptone Soya Broth (TSB) (Oxoid, Hampshire, UK). Bacteria were then chilled on ice for 1 h, centrifuged at 4000× *g* for 10 min at 4 °C and washed three times with ice-cold sterile deionized water. The final pellet following these washes was suspended in ice-cold 10% glycerol and aliquoted for −80 °C storage. 100 ng of pBAV-MRSA-NL DNA was added to thawed aliquots and incubated for 30 min at room temperature prior to electroporation. Electroporation was performed using a MicroPulser Plus (1.8 kV voltage, 1 pulse, 2.5 ms time constant) with 0.2 cm cuvettes (Bio-Rad, Marnes-la-Coquette, France). Cells were recovered in B2 medium (10 g/L peptone, 25 g/L yeast extract, 25 g/L NaCl, 1 g/L K_2_HPO_4_, pH 7.5) and spread on TSB agar with 50 µg/mL kanamycin (Sigma, St. Louis, MO, USA). Transformants were isolated and confirmed by expression of NanoLuc. Colonies were grown for 3 h in TSB with kanamycin before being tested. A mixture of 10 µL of culture, 50 µL of NanoGlo buffer, 15 µL Renilla lysis buffer and 1 µL of NanoGlo substrate (Promega, Madison, WI, USA) was prepared and analyzed using a GloMax Navigator (Promega, Madison, WI, USA).

NanoLuc-positive cultures of transformed RN4220 were grown to early log-phase and infected with either MP115 or ISP at a MOI of 0.1 and incubated for 3 h at 37 °C with shaking at 225 rpm. The phage lysate was centrifuged to remove cell debris, filtered through a Whatman Puradisc filter, 0.45 µM pore size (GE Health, Pittsburgh, PA, USA) and finally buffer-exchanged into TMS using a 100 K MWCO protein concentrator (Pierce). Limiting dilution enrichment was then performed to increase the frequency of recombinants prior to isolation by plaque screening on semi-solid agar. Individual plaques were isolated using a sterile pipet tip and mixed with 100 µL of TMS buffer. 10 µL of this suspension was used to infect 100 µL of strain 12600 in TSB for 2 h at 37 °C. After infection, 50 µL of NanoGlo buffer, 15 µL Renilla lysis buffer and 1 µL of NanoGlo substrate was added to each well, before being assessed on a GloMax Navigator. A strong bioluminescent signal is expected to be detected from wells containing NanoLuc-positive recombinants. Once identified, these positive wells were filtered, diluted and used to infect the next passage. Verification of NanoLuc insertion by PCR was performed using the Q5 DNA polymerase (New England Biolabs, Ipswich, MA, USA) according to manufacturer’s instructions. The primer sequences 5’-CTGACCCTACTAATGCATCAGAG-3’ and 5’-CAGGAGTGATTCCATAACCAGTT-3’ flank the targeted insertion site and confirmed the expected shift of approximately 600 bp following homologous recombination. Purity of each recombinant was achieved by repeated plaque isolation and three successive passages yielded plaques that were 100% positive for bioluminescence. The use of three consecutive rounds of single plaque isolation has been used by others to guarantee the purity of bacteriophage stocks [30].

Following purification, whole genome sequencing was performed to confirm production of the anticipated recombinant. To obtain phage DNA, at least 5 × 10^9^ pfu were heated at 90 °C for 3 min. Purification was achieved through three phenol/chloroform extractions. Following this, the aqueous phase was mixed with 0.1 volume of 3 M sodium acetate and 2.5 volumes of absolute ethanol. DNA was precipitated overnight at −80 °C, pelleted and washed twice with 70% ethanol. Finally, purified DNA was pelleted, air-dried and resuspended in deionized water. Whole genome sequencing was performed with this DNA by Laragen Inc. (Culver City, CA, USA) using Illumina MiSeq. Sequencing confirmed the anticipated insertion site and overall generation of the desired recombinants.

### 2.4. In Vitro Phage Detection Assays—Sensitivity, Inclusivity and MSSA Exclusivity

Overnight cultures were diluted in BHI broth and 135 µL transferred to two wells of a 96-well strip plate (Griener Bio-One GmbH, Frickenhausen, Germany) to obtain the desired colony forming units (CFU) per well. An additional two wells consisting of only 135 µL of BHI broth were utilized to determine the medium background. One well for each sample served as a control well and received 15 µL of BHI broth. The other well served as a selective well, receiving 15 µL of BHI broth containing 22 µg/mL cefoxitin (Alfa Aesar, Ward Hill, MA, USA). The selective well had a final concentration of 2.2 µg/mL cefoxitin. When indicated, actual CFU for each sample was confirmed by plate counting on BHI agar. The 96-well strip plate was sealed with cover film (Thermo Fisher Scientific, Rochester, NY, USA) and incubated for 4 h at 37 °C to facilitate enrichment and selection. A phage cocktail was prepared in LB broth (Gibco, Grand Island, NY, USA) and contained both engineered phages at 1.6 × 10^8^ PFU per mL each. 10 µL of this phage cocktail was added to each well and mixed by pipetting before being covered once again with film. The plate was incubated for 2 h at 37 °C to promote phage infection and production of luciferase in the presence of MRSA. 65 µL of detection solution consisting of 50 µL NanoGlo Buffer, 15 µL Renilla lysis buffer and 1 µL of NanoGlo substrate was added to each well and mixed by pipetting. Samples were read using a GloMax Navigator with a 3 min wait time and 1 s integration. Results were evaluated with a cut-off of 600 relative light units (RLU), which is approximately three times the background observed with medium alone.

### 2.5. In Vitro Phage Detection Assay—Non-Staphylococcus aureus Exclusivity and Bacterial Interference

Overnight cultures of competitor organisms were diluted in BHI broth and 125 µL transferred to four wells of a 96-well strip plate to obtain desired CFU per well. An additional four wells consisting of only 125 µL of BHI broth were utilized to determine medium background and baseline signal of MRSA (BAA-1720). Two wells of each sample were assigned to exclusivity tests, while the other two wells were used to assess bacterial interference. For exclusivity, 10 µL of BHI broth was added to both wells while 10 µL of BHI broth containing MRSA was added to bacterial interference wells. For each condition, one well served as a control well and received an additional 15 µL of BHI broth while the other served as a selective well and received 15 µL of BHI broth containing 22 µg/mL cefoxitin. Enrichment, phage infection and CFU determination were then performed as previously described in Section 2.4.

### 2.6. Nasal Swab Phage Detection—Endogenous Samples, MRSA Spike and Autoluminescence

The BBL CultureSwab Liquid Stuart Double swab (Becton Dickinson and Company, Sparks, MD, USA) was used in this study. Rayon nasal swabs were self-collected from volunteers who were instructed to insert the swab into one nostril, rotate at least five times and repeat with the same swab in the second nostril. Written consent was obtained from individuals participating in internal collections. Prior to processing, specimens were stored overnight at 4 °C. To evaluate endogenous nasal samples, one swab was eluted by vortexing for 15 s in 1 mL of BHI broth. 135 µL of this nasal elutant was added to two wells of a 96-well strip plate. These wells were assessed in the same manner as the 135 µL diluted cultures described in Section 2.4. 

A reference method using both direct plating and enriched culture was employed to identify true MRSA colonization. For direct plating, 135 µL of nasal elutants used in the screen was plated on MRSA Select II agar. For the enriched culture method, one swab was placed in 3 mL of TSB with 6.5% NaCl (Fisher Scientific, Geel, Belgium) and grown overnight at 37 °C with shaking at 250 rpm. The next day the culture was streaked on MRSA Select II agar. In both cases, manufacturer’s instructions were followed to identify the presence or absence of MRSA colonization. Swabs were considered MRSA positive if either method (direct plating or enriched culture) yielded a positive result on selective agar.

The capacity for MRSA detection in nasal matrix was assessed by spiking diluted cultures of MRSA into nasal elutants. To this end, 125 µL of nasal elutants was added to two wells of a 96-well strip plate for each sample. Both wells received 10 µL of a diluted MRSA culture. 40 unique nasal samples were assessed with eight samples assigned per MRSA strain tested (BAA-1707, BAA-1717, BAA-1720, BAA-1763, BAA-1766). As a control, 10 µL of each MRSA strain was also spiked into 125 µL of BHI broth. After spiking, the two wells were assessed in the same manner as the 135 µL diluted cultures described in Section 2.4. 

Autoluminescence of each nasal sample was assessed by mixing each sample with detection solution without the source of luciferase (phage cocktail). To accomplish this, 135 µL of each nasal elutant was combined with 25 µL of BHI broth in a 96-well strip plate. 65 µL of detection solution was then added to each well and pipetted to mix. The plate was read on a luminometer as previously described in Section 2.4.

## 3. Results

### 3.1. Sensitivity and Inclusivity of the MRSA Screen In Vitro

A novel bacteriophage-based MRSA screen was developed to provide actionable results in a single 8 h shift. Two wells of a 96-well plate were utilized for each sample—one control well and one selective well. The selective well is used for MRSA determination and contains an MRSA selective agent, cefoxitin, while the control well contains only a bacterial culture medium and primarily gauges phage performance during assay development. Cefoxitin has been found to be a superior choice for phenotypic identification of MRSA in disc diffusion and agar dilution assays [31,32,33]. The samples were enriched in these wells for 4 h, which facilitated recovery, growth and selection of resistant bacteria. Following this, a 2 h infection period with recombinant luciferase-encoding bacteriophage was performed. Production of luciferase, indicative of successful viral infection, is measured by detection of emitted light with a luminometer after the addition of substrate. 17 diverse MRSA strains were evaluated using this method at a starting target of 10, 100 or 1000 colony forming units (CFU) in triplicate wells (Table 1). CFU determined from plate counts and relative light units (RLU) values are provided (Table S1). A positive result was determined based upon a cutoff of 600 RLU. This cutoff is approximately three times the background observed with culture media alone. A positive result was obtained for 51 of 51 wells tested (100%) at both 100 and 1000 CFU per well in control conditions. At 10 CFU per well, 48 of 51 wells (94.1%) were positive. Three unique strains of MRSA were positive in only two of three wells at 10 CFU. These results highlight the ability of the phage cocktail to recognize diverse MRSA isolates. When cefoxitin was included for MRSA determination, a positive signal could still be detected for 51 of 51 wells (100%) at 1000 CFU per well and 48 of 51 wells (94.1%) at 100 CFU per well. The inability to detect BAA-42, also known as HDE288, at 100 CFU under selection is not entirely unexpected. This strain belongs to an “archaic clone” of MRSA, associated with low-level and heterogeneous methicillin resistance [34]. 44 of 51 selective wells (86.3%) remained positive with only 10 CFU. A limit of detection was determined for each strain based upon the lowest CFU with 100% detection in both control and selective wells. 13 of the 17 MRSA strains tested could be reliably detected at 10 CFU per well, while three required 100 CFU per well. BAA-42 was the only strain to require greater than 100 CFU per well for consistent positive detection with MRSA selection. Overall, these results demonstrate the ability of this screen to detect the presence of genetically diverse MRSA strains at low bacterial burdens.

### 3.2. Exclusivity and Specificity of the MRSA Screen In Vitro 

In addition to sensitive MRSA detection, a successful screen must also demonstrate the ability to exclude a majority of MSSA strains. Five well-characterized strains of MSSA were evaluated with this method at 100, 1000 and 10,000, target CFU in triplicate wells (Table 2). CFU determined from plate counts and RLU values are provided (Table S2). As expected, MSSA strains were positive in 100% of control wells at 100, 1000 and 10,000 CFU. The inclusion of cefoxitin in the selective wells resulted in significant reduction of positive results. At 100 CFU, 0 of 15 (0%) selective wells were positive, while only 1 well of 15 (6.7%) was positive at 1000 and 10,000 CFU. These results support the ability of this method to discriminate against most MSSA strains.

Beyond MSSA, the exclusivity of the MRSA screen was evaluated in vitro against a panel of 40 strains, encompassing 21 unique genera and 38 distinct species (Table 3). CFU determined from plate counts and RLU values are provided (Table S3). CFU for each exclusivity strain was greater than 1500 CFU per well (median CFU of 15,950). When assessing specificity, 6 of 40 (15.0%) strains were positive in the control well. Positive signal in this condition is the result of cross-reactivity of the phage cocktail and was observed with *Staphylococcus* and *Bacillus* species. Many *Staphylococcus aureus* phages have been demonstrated to be polyvalent, lysing both coagulase-positive and negative staphylococcal species [35,36,37]. Adsorption of staphylococcal phages by *Bacillus* species has previously been reported and may be associated with similarities in their cell wall teichoic acid (WTA) [38,39]. Despite this cross-reactivity, 0 of 40 strains were positive in the selective condition and would not have resulted in false positives for MRSA. These results demonstrate the specificity of the phage cocktail used in this study and the exclusivity of the overall assay. 

The ability of the screen to detect low numbers of MRSA in the presence of excess competitor burdens was assessed. To this end, approximately 50 CFU of MRSA was combined with at least a 20-fold excess of each strain from the exclusivity panel (Table 3). CFU determined from plate counts and RLU values are provided (Table S3). In the presence of competitor species, 39 of 40 (97.5%) and 40 of 40 (100%) wells were positive in the control and selective conditions, respectively. *Streptococcus pneumoniae* inhibited detection in the control conditions when tested at 100-fold excess. This is not surprising, given the known antagonism between these species both in vitro and in vivo [40,41,42,43]. Critically, this effect was lost in the presence of cefoxitin (MRSA selective condition) and would thus not result in a false negative for MRSA. These data support the ability of this screen to detect low-levels of MRSA in environments containing excess competing organisms.

### 3.3. Screen Performance among Circulating Staphylococcus aureus Clinical Isolates In Vitro

MRSA isolates from human clinical specimens were obtained internally from three geographically distinct clinical microbiology labs in the United States (Burlington NC, USA, Phoenix AZ, USA and Raritan NJ, USA). MSSA isolates were obtained in a similar fashion from one site (Burlington, NC, USA). MRSA or MSSA identification was confirmed by plating on selective chromogenic agar. A total of 390 clinical MRSA strains were isolated from unique specimens and evaluated with the MRSA screen. RLU and CFU values for each strain are provided (Table S4). The median burden of MRSA tested was 47 CFU per well. 388 of 390 clinical MRSA strains (99.5%) were positively detected in the control well (Table 4). Under cefoxitin selection, 381 of 390 (97.7%) clinical MRSA strains were positive and identified by the screen as MRSA. Clinical MSSA strains were tested for exclusion at higher burdens, either 10- or 100-times MRSA levels (500 and 5000 CFU respectively). 122 of 123 (99.2%) clinical MSSA strains were positively detected in the control condition of either inoculum. In selective wells, however, positive signal from 500 CFU dropped to 8 of 123 (6.5%) MSSA strains. At approximately 5000 CFU per well, this rate of false positives increased to 21 of 123 (17.1%) strains. This suggests that, while most MSSA strains will be negative, some may overwhelm selection at high burdens and result in false positives. Critically, of 513 tested clinical *Staphylococcus aureus* isolates, 510 (99.4%) were positive in the control condition. This continues to support the notion that the phage cocktail utilized in this system yields broad-host-range coverage. Overall, these results show the capability of this screen to successfully recognize and detect the vast majority of clinical MRSA strains, while excluding many clinical MSSA strains.

### 3.4. Specificity and Screen Performance with Human Nasal Swabs 

Anterior nare specimens were self-collected by 40 adult human volunteers using a rayon swab. Previous studies have confirmed the efficacy of self-collection for the detection of MRSA colonization [44,45]. Prior to processing, specimens were stored over-night at 4°C to mimic possible sample shipping conditions. A reference method using both direct plating and enriched culture was employed to identify true MRSA colonization. All 40 human nasal specimens were negative by both reference methods and were determined to lack MRSA colonization (Table 5). This lack of detection among 40 individuals is not surprising, as the rate of MRSA colonization among healthy adults has been estimated at less than 2% [46]. To perform the screen with these specimens, the swab was eluted into bacterial culture media and added to wells with (selective) or without (control) cefoxitin. A positive result in the selective condition is considered to be a MRSA positive result. The control condition is not required or utilized for MRSA determination but was included to demonstrate the effectiveness of selection. A positive result was anticipated in most control wells due to the high nasal colonization rates of staphylococcal species and the cross-reactivity previously described with the phage cocktail [47,48]. In agreement with expectations, 36 of 40 (90%) samples were positive in the control well. RLU values for endogenous samples are provided (Table S5). 36 of 40 specimens (90.0%) were negative for MRSA detection and agreed with the reference method. False positives were identified in four samples, with a median RLU signal of less than five times the signal cutoff. All nasal samples were negative when tested directly with luciferase substrate, indicating that non-specific autoluminescence was not a significant source of false positives (Table S5). The exact mechanism behind the false positive signal in these samples remains unknown but could potentially be linked to methicillin-resistant coagulase-negative staphylococci. Additionally, some MSSA strains were previously observed to result in false positives at high bacterial burdens (Table 4). Overall, the majority (90%) of MRSA-negative samples could be successfully screened out by this method. 

In order to determine if this method could successfully detect MRSA in nasal matrix, five well-characterized MRSA strains were spiked into the elutants from the previously described 40 non-colonized nasal swabs. RLU and CFU values for each sample are provided (Table S5). The median burden of a MRSA spike was 87 CFU per well, which is equivalent to approximately 650 CFU per swab. 40 of 40 (100%) MRSA spiked samples were positive in both the control and selective conditions (Table 5). The lack of any false negatives with spiked specimens suggests the absence of assay inhibitors in these individuals. The successful detection of five unique MRSA strains when spiked into these samples at low burdens supports the efficacy of bacteriophage-based screening in nasal matrix. 

## 4. Discussion

Nasal carriage of MRSA predisposes individuals to infection and significantly increases their risk of complications associated with morbidity and mortality [10,12,49]. To mitigate this risk, various forms of surveillance and decolonization have been recommended [14,15]. In support of these initiatives, novel methods of MRSA detection may be beneficial if they are sufficiently sensitive, rapid and cost-effective. The purpose of this work was to generate the first MRSA luciferase phage reporter assay and in utilizing this inexpensive culture-based approach, achieve sensitive and rapid detection of MRSA from nasal swabs.

The diagnostic screen developed in this study was capable of identifying MRSA strains from diverse genetic backgrounds in approximately 6 h (Table 1). For the vast majority of MRSA strains, successful detection required the presence of only 10 to 100 CFU per well, approximately equivalent to 75 to 750 CFU per nasal swab. This limit of detection is similar to previously described PCR-based screens [16,19]. The median burden of MRSA recovered from nasal swabs of carriers has been found to be greater than 10,000 CFU [50]. Additionally, individuals with high burdens of nasal colonization are more likely to carry MRSA at multiple body sites and be vectors for transmission [51,52]. The sensitivity of this assay thus appears well-suited to address the expected burden from clinical nasal specimens whether the goal is to eliminate MRSA carriage or limit patient to patient spread. 

The performance of luciferase reporter phage assays is highly dependent on the selection of bacteriophage. This MRSA diagnostic screen utilized NanoLuc-expressing recombinants of two phage, ISP and MP115, which are members of the *Myoviridae* family of large lytic staphylococcal bacteriophages [27,28,53]. These phages bind to the host surface primarily through the highly conserved WTA backbone, resulting in broad-host-range capabilities [54]. The use of this core *S. aureus* structure as a binding site facilitates the infection of strains regardless of glycosylation, alanylation and likely other WTA modifications [55]. Thus, while formally possible, resistance to the phages used in this assay through alterations in this receptor is not expected to emerge. While WTA modifications are well tolerated by these phages, *S. aureus* mutants lacking WTA are thought to be resistant to all or at least most, staphylococcal phages [56]. Although resistant WTA-deficient mutants are hypothetically possible, previous studies have revealed that WTA is required for both nasal colonization and methicillin resistance [57,58,59,60]. Generally, the loss of WTA also results in a fitness cost in vivo and overall decrease in virulence [61,62]. It is reasonable to expect that all current and future MRSA strains involved in nasal carriage will possess the receptor targeted by this screen. This is further supported by the positive phage signal detected for 99.5% of clinical MRSA isolates tested in this study (Table 4).

In our study of 513 *Staphylococcus aureus* clinical strains, two isolates of MRSA (BNC 159 and PHX 079) and one isolate of MSSA (MSSA 090) failed to generate a positive signal in the control condition (Table 4). One of these isolates (PHX 079) appeared to have a growth defect in culture (data not shown). Poor growth during the enrichment period could have contributed to the inability to reliably detect this MRSA strain. Failure to detect BNC 159 and MSSA 090 may be associated with phage resistance through a variety of potential pathways [63]. While elucidating this resistance mechanism was outside the scope of this work, future studies on these isolates may be beneficial. 

The combination of a luciferase reporter phage and antibiotic has been used previously to detect drug-resistance [4]. This approach was employed in the current study utilizing cefoxitin to restrict the viability and growth of non-MRSA. The results herein support this selection, as only 6.5% of clinical MSSA strains were positive when tested at approximately 500 CFU per well (Table 4). Critically, cefoxitin did not interfere with MRSA detection, as 97.7% of clinical MRSA strains remained positive in selective wells at approximately 50 CFU per well. This selective agent also proved beneficial in restricting the false positives from several species of *Bacillus* and coagulase-negative staphylococci, while also preventing interference from *Streptococcus pneumoniae* (Table 3). Despite the high rate of detection of clinical MRSA, some strains did yield false-negative results in the presence of cefoxitin. Since clinical MRSA strains were evaluated at particularly low burdens in this study, it is plausible that these strains express low-level resistance or heteroresistance. Such strains may present a limit of detection greater than 100 CFU per well, similar to that found for BAA-42 (Table 1).

Regarding performance with nasal swabs, 90.0% of MRSA-negative samples gave a negative test result under selection and agreed with the reference method (Table 5). False positives were thus detected in 10% of nasal elutants. These false positives may originate from three sources. First, autoluminescence may occur but was ruled out in these samples by demonstrating a requirement for added luciferase. (Table S5). Second, high burdens of certain MSSA strains may result in false positives (Table 4). Finally, some cross-reacting species of coagulase-negative staphylococci can become methicillin-resistant through the same resistance mechanism as MRSA [64]. These species could potentially contribute to the weak false MRSA positives observed in four samples. Future studies are warranted to address these potential explanations.

One limitation of our study is the lack of endogenous MRSA positive nasal swabs. To compensate for this deficiency, nasal elutants were spiked with one of five MRSA strains (Table 5). Positive detection of low MRSA burdens in nasal matrix was achieved in 100% of spiked samples. Importantly, this indicates that successful bacteriophage infection and luciferase production is capable of occurring in the nasal matrix. Furthermore, this reveals that the negative control wells seen previously in 10% of endogenous samples were not the result of assay inhibitors. Overall, the results strongly suggest that MRSA carriage, when present, would be detected in nasal specimens. 

This study presents the development and evaluation of a novel bacteriophage-based MRSA diagnostic screen. This assay is a member of a new generation of luciferase reporter phage systems utilizing NanoLuc to sensitively detect target species. The method proved to be highly inclusive and, when combined with cefoxitin selection, discriminated against the majority of non-resistant strains. Moreover, the screen was capable of identifying low burdens of MRSA in nasal samples with no evidence of problematic interference. With MRSA calls made in 6 h, actionable results would be available in a single work shift. Ultimately, these data support the notion that this diagnostic screen may be a promising new tool for the detection of MRSA colonization from nasal swabs.

## Figures and Tables

**Table 1:** In Vitro sensitivity and inclusivity.

Strain ID ^1^	SCCmec ^2^	PFGE ^2^	# of Positive ^3^ Control			# of Positive ^3^ Selective			LoD ^4^
			10	100	1000	10	100	1000	CFU
BAA-44	I	Iberian	3/3	3/3	3/3	3/3	3/3	3/3	10
BAA-41	II	USA 100	2/3	3/3	3/3	3/3	3/3	3/3	100
BAA-1761	II	USA 100	3/3	3/3	3/3	3/3	3/3	3/3	10
BAA-1720	II	USA 200	3/3	3/3	3/3	3/3	3/3	3/3	10
33592	III	ST239	3/3	3/3	3/3	3/3	3/3	3/3	10
BAA-1717	IV	USA 300	3/3	3/3	3/3	3/3	3/3	3/3	10
BAA-1683	IV	USA 400	3/3	3/3	3/3	3/3	3/3	3/3	10
BAA-1707	IV	USA 400	2/3	3/3	3/3	0/3	3/3	3/3	100
BAA-1763	IV	USA 500	3/3	3/3	3/3	3/3	3/3	3/3	10
BAA-1754	IV	USA 600	3/3	3/3	3/3	3/3	3/3	3/3	10
BAA-1768	IV	USA 800	3/3	3/3	3/3	2/3	3/3	3/3	100
BAA-1747	IV	USA 1000	3/3	3/3	3/3	3/3	3/3	3/3	10
BAA-1764	IV	USA 1100	3/3	3/3	3/3	3/3	3/3	3/3	10
BAA-1766	V	USA 700	3/3	3/3	3/3	3/3	3/3	3/3	10
BAA-2094	V	WA-MRSA	3/3	3/3	3/3	3/3	3/3	3/3	10
BAA-42	VI	USA 800	2/3	3/3	3/3	0/3	0/3	3/3	1000
BAA-2313	XI	CC130	3/3	3/3	3/3	3/3	3/3	3/3	10
Total number of positives (%):			48/51 (94.1)	51/51 (100)	51/51 (100)	44/51 (86.3)	48/51 (94.1)	51/51 (100)	

^1^ Strain ID corresponds to American Type Culture Collection (ATCC) catalog numbers. ^2^ SCCmec Type and pulse field gel electrophoresis (PFGE) was available from the (ATCC). ^3^ Number (#) of positive wells were defined based on a signal cutoff of 600 relative light units (RLU). ^4^ Limit of detection (LoD) was defined as the lowest starting colony forming units (CFU) that displayed 100% positive results in both control and selective wells.

**Table 2:** In Vitro discrimination of methicillin-susceptible *Staphylococcus aureus* (MSSA).

Strain ID ^1^	Type	# of Positive ^2^ Control			# of Positive ^2^ Selective		
		100	1000	10000	100	1000	10000
6538	MSSA	3/3	3/3	3/3	0/3	0/3	0/3
12600	MSSA	3/3	3/3	3/3	0/3	1/3	1/3
14775	MSSA	3/3	3/3	3/3	0/3	0/3	0/3
25923	MSSA	3/3	3/3	3/3	0/3	0/3	0/3
29213	MSSA	3/3	3/3	3/3	0/3	0/3	0/3
Total number of positives (%):		15/15 (100)	15/15 (100)	15/15 (100)	0/15 (0.0)	1/15 (6.7)	1/15 (6.7)

^1^ Strain ID corresponds to ATCC catalog numbers. ^2^ Number (#) of positive wells were defined based on a signal cutoff of 600 RLU.

**Table 3:** In Vitro exclusivity and assay performance with bacterial competitors.

Genus	Species	Strain ID ^1^	Exclusivity ^3^ (Competitor Only)		Bacterial Interference ^4^ (Competitor + MRSA)	
			Control	Selective	Control	Selective
*Staphylococcus*	*epidermidis*	14990	Negative	Negative	Positive	Positive
		700583	Positive	Negative	Positive	Positive
	*haemolyticus*	29970	Positive	Negative	Positive	Positive
		700564	Negative	Negative	Positive	Positive
	*hominis*	27844	Negative	Negative	Positive	Positive
	*lugdunensis*	49576	Negative	Negative	Positive	Positive
	*saprophyticus*	15305	Positive	Negative	Positive	Positive
	*warneri*	49454	Positive	Negative	Positive	Positive
*Bacillus*	*licheniformis*	9789	Negative	Negative	Positive	Positive
	*pumilus*	700814	Positive	Negative	Positive	Positive
	*subtilis*	6051	Positive	Negative	Positive	Positive
*Citrobacter*	*braaki*	51113	Negative	Negative	Positive	Positive
	*freundii*	8090	Negative	Negative	Positive	Positive
	*koseri*	25408	Negative	Negative	Positive	Positive
*Enterococcus*	*faecalis*	19433	Negative	Negative	Positive	Positive
	*faecium*	19434	Negative	Negative	Positive	Positive
*Klebsiella*	*oxytoca*	43165	Negative	Negative	Positive	Positive
	*pneumoniae*	4352	Negative	Negative	Positive	Positive
*Listeria*	*innocua*	51742	Negative	Negative	Positive	Positive
	*ivanovii*	19119	Negative	Negative	Positive	Positive
	*monocytogenes*	19115	Negative	Negative	Positive	Positive
	*welshimeri*	35897	Negative	Negative	Positive	Positive
*Proteus*	*mirabilis*	43071	Negative	Negative	Positive	Positive
	*vulgaris*	33420	Negative	Negative	Positive	Positive
*Shigella*	*flexneri*	12022	Negative	Negative	Positive	Positive
	*sonnei*	9290	Negative	Negative	Positive	Positive
*Streptococcus*	*pneumoniae*	6303	Negative	Negative	Negative	Positive
	*pyogenes*	12202	Negative	Negative	Positive	Positive
*Acinetobacter*	*baumannii*	19606	Negative	Negative	Positive	Positive
*Edwardsiella*	*tarda*	15947	Negative	Negative	Positive	Positive
*Enterobacter*	*kobei*	BAA-260	Negative	Negative	Positive	Positive
*Escherichia*	*coli*	25922	Negative	Negative	Positive	Positive
*Hafnia*	*alvei*	13337	Negative	Negative	Positive	Positive
*Moraxella*	*catarrhalis*	25238	Negative	Negative	Positive	Positive
*Morganella*	*morganii*	25830	Negative	Negative	Positive	Positive
*Pluralibacter*	*gergoviae*	33028	Negative	Negative	Positive	Positive
*Pseudomonas*	*aeruginosa*	27853	Negative	Negative	Positive	Positive
*Salmonella*	*enterica*	S492	Negative	Negative	Positive	Positive
*Serratia*	*marcescens*	13880	Negative	Negative	Positive	Positive
*Yersinia*	*enterocolitica*	23715	Negative	Negative	Positive	Positive
Total number of positives ^2^ (%):			6/40 (15.0)	0/40 (0.0)	39/40 (97.5)	40/40 (100)

^1^ Strain ID corresponds with ATCC catalog number for all strains except *Salmonella enterica* strain S492. ^2^ Positive wells were defined based on a signal cutoff of 600 RLU. ^3^ For exclusivity, each competitor strain was assessed alone at greater than 1500 CFU per well. ^4^ For bacterial interference, MRSA (BAA-1720) was added at approximately 50 CFU per well while indicated competitor strains were added in excess (at least 20-fold).

**Table 4:** Performance of methicillin-resistant *Staphylococcus aureus* (MRSA) screen with clinical *Staphylococcus aureus.*

	Clinical MRSA			Clinical MSSA		
	CFU ^2^	Control	Selective	CFU ^3^	Control	Selective
Number of positives ^1^ (%):	50	388/390 (99.5)	381/390 (97.7)	500	122/123 (99.2)	8/123 (6.5)
				5000	122/123 (99.2)	21/123 (17.1)

^1^ Positive wells were defined based on a signal cutoff of 600 RLU. ^2^ The median CFU tested for clinical MRSA strains was 47 CFU per well. The burden for each strain can be found in the supplement. ^3^ The median CFU per well tested for clinical MSSA was 850 CFU for “500” and 8500 CFU for “5000.”

**Table 5:** Screen performance with non-colonized nasal swabs.

	Endogenous Nasal Samples ^2^ (Elutant Only)			Detection in Nasal Matrix ^3^ (Elutant + MRSA)	
	Control	Selective	Reference ^4^	Control	Selective
Number of positives ^1^ (%):	36/40 (90.0)	4/40 (10.0)	0/40 (0.0)	40/40 (100)	40/40 (100)

^1^ Positive wells were defined based on a signal cutoff of 600 RLU. ^2^ Nasal swabs were eluted in bacterial culture media and assayed directly. ^3^ Nasal elutants were spiked with one of five MRSA strains at approximately 100 CFU per well before testing. ^4^ A combination of direct plating and enriched cultures was employed as a reference method using MRSA Select II agar.

## Supplementary Materials

The following are available online at https://www.mdpi.com/1999-4915/12/6/631/s1. Table S1: CFU and RLU for In Vitro Sensitivity and Inclusivity (Table 1). Table S2: CFU and RLU for In Vitro Discrimination of MSSA (Table 2). Table S3: CFU and RLU for Exclusivity and Assay Performance with Bacterial Competitors (Table 3). Table S4: CFU and RLU for MRSA Screen with Clinical *Staphylococcus aureus* (Table 4). Table S5: CFU and RLU for Nasal Swabs: Endogenous, MRSA Spike and Autoluminescence (Table 5).
