# Supplementary material for: Development and Evaluation of a Sensitive Bacteriophage-Based MRSA Diagnostic Screen
Source: Viruses. 2020 Jun 11;12(6):631. doi: 10.3390/v12060631 (PMC7354448; doi:10.3390/v12060631)
Supplement: Supplementary file 1 [file viruses-12-00631-s001.pdf]

Table S1. CFU and RLU for *in vitro* sensitivity and inclusivity (Table 1)

Strain	Target CFU	CFU[1]	Control RLU			Selective RLU		
			Well 1	Well 2	Well 3	Well 1	Well 2	Well 3
BAA-44	10	3	14110	66460	31470	18440	56720	25170
BAA-44	100	28	476000	611800	683000	476900	492800	603300
BAA-44	1000	280	9183000	9172000	7862000	5249000	5136000	4743000
BAA-41	10	5	3969	176	18070	7585	3113	9434
BAA-41	100	49	508600	612900	346200	113600	159700	159900
BAA-41	1000	529	14780000	15850000	17730000	5131000	5079000	5018000
BAA-1761	10	5	866	30140	49990	8856	2533	4631
BAA-1761	100	51	267900	333500	306500	33880	47440	60150
BAA-1761	1000	510	6356000	4524000	5751000	1000000	1143000	1010000
BAA-1720	10	7	83630	74490	108800	52380	49230	32800
BAA-1720	100	71	811100	963600	1126000	169100	238100	288100
BAA-1720	1000	705	10260000	10190000	10290000	2976000	3258000	3571000
33592	10	11	5623	15710	6434	1418	2601	2513
33592	100	112	86640	73700	63510	18660	18710	25040
33592	1000	1115	828700	903400	866900	210800	193400	197000
BAA-1717	10	10	261500	240200	339200	43620	2636	51360
BAA-1717	100	98	4324000	4548000	3875000	729200	690700	664100
BAA-1717	1000	1022	32010000	30010000	33700000	9901000	9117000	11980000
BAA-1683	10	10	62430	204100	13640	100500	143500	86780
BAA-1683	100	101	1010000	1119000	1213000	1031000	831200	970100
BAA-1683	1000	1010	11710000	11380000	12080000	11840000	9533000	10220000
BAA-1707	10	3	158	505900	108200	166	166	156
BAA-1707	100	31	1738000	1349000	1459000	701000	942200	1476000
BAA-1707	1000	278	32470000	33450000	33850000	16860000	20560000	22670000
BAA-1763	10	7	9399	14320	13450	933	3832	4106
BAA-1763	100	68	250700	257700	206000	16340	37160	32910
BAA-1763	1000	675	1317000	1411000	1461000	289700	326600	304100
BAA-1754	10	14	335700	460500	404900	186800	251300	195400
BAA-1754	100	137	4676000	4059000	4630000	2619000	1824000	2948000
BAA-1754	1000	1249	39970000	43020000	45350000	23920000	23090000	23370000
BAA-1768	10	1	1101000	1038000	346000	4526	3589	172
BAA-1768	100	14	8948000	8918000	5671000	62360	53840	65240
BAA-1768	1000	135	62560000	71830000	69130000	1195000	1206000	913700
BAA-1747	10	11	21440	26280	33830	9141	15180	13110
BAA-1747	100	105	90310	138600	180700	88070	77420	80040
BAA-1747	1000	1050	676700	698800	757100	335500	387200	418900
BAA-1764	10	14	332500	267300	96030	169000	109000	109600
BAA-1764	100	137	2954000	3075000	2388000	1123000	1096000	1138000
BAA-1764	1000	1365	21630000	20130000	24990000	11590000	10320000	10430000
BAA-1766	10	6	90370	262800	200800	7756	9880	1067
BAA-1766	100	58	1641000	1763000	1984000	155500	95160	223400
BAA-1766	1000	575	30650000	27920000	31810000	2236000	1752000	2099000
BAA-2094	10	9	424200	545500	273900	127600	166500	117900
BAA-2094	100	86	4371000	4259000	4753000	1274000	830900	1051000
BAA-2094	1000	1172	42320000	45310000	40370000	9401000	9215000	10580000
BAA-42	10	3	646	786	390	165	145	247
BAA-42	100	30	5895	7651	6028	328	457	390
BAA-42	1000	295	30870	42400	34910	3511	2770	4244
BAA-2313	10	8	449000	323100	603700	39570	77110	16000
BAA-2313	100	75	6515000	6700000	6655000	289200	192400	141400
BAA-2313	1000	750	53120000	54760000	56490000	4115000	3926000	3840000
BHI[2]	-	-	148	126	162	115	141	152

[1] CFU were determined by plate counting (in duplicate) for samples with a target of 100 CFU per well and calculated from dilutions for samples with a target of 10 and 1000 CFU. [2] BHI broth was used in place of bacterial culture to identify assay background.

Table S2. CFU and RLU for *in vitro* discrimination of MSSA (Table 2)

Strain	Target CFU	CFU[1]	Control RLU			Selective RLU		
			Well 1	Well 2	Well 3	Well 1	Well 2	Well 3
6538	100	43	847900	1997000	1008000	130	146	142
6538	1000	425	15860000	14890000	15170000	148	133	153
6538	10000	4250	151500000	153200000	158100000	183	195	223
12600	100	192	3399000	3173000	4102000	136	192	131
12600	1000	1920	43220000	39030000	38470000	155	775	143
12600	10000	19200	125700000	126800000	147500000	232	653	160
14775	100	166	5037000	5107000	5413000	140	142	151
14775	1000	1655	66210000	60400000	64720000	110	141	142
14775	10000	16550	97360000	95240000	96340000	123	131	150
25923	100	65	1977000	2553000	1673000	146	116	161
25923	1000	645	25380000	22220000	25040000	121	128	152
25923	10000	6450	58650000	60290000	65420000	125	143	157
29213	100	174	83090	103900	99960	121	130	131
29213	1000	1740	308400	296400	273700	118	151	130
29213	10000	17400	700800	777800	784500	133	268	132
BHI[2]	-	-	181	115	-	125	111	-

[1] CFU were determined by plate counting (in duplicate) for samples with a target of 100 CFU per well and calculated from dilutions for samples with a target of 10 and 1000 CFU. [2] BHI broth was used in place of bacterial culture to identify assay background.

Table S3. CFU and RLU for exclusivity and assay performance with bacterial competitors (Table 3)

Genus	Species	Strain ID	Competitor CFU[1]	RLU for Exclusivity[3] (Competitor only)		RLU for Bacterial Interference[4] (Competitor + MRSA)		
				Control	Selective	MRSA CFU[1]	Control	Selective
Staphylococcus	*epidermidis*	14990	62300	256	152	52	199300	119800
		700583	36600	317400	170	52	403700	166600
	haemolyticus	29970	14400	1759000	138	52	2447000	154000
		700564	30000	176	223	52	182500	90610
	hominis	27844	9800	170	175	52	467100	190200
	lugdunensis	49576	16100	118	270	71	362300	90950
	saprophyticus	15305	16650	6282	250	52	420100	231500
	warneri	49454	9750	41750000	455	53	45850000	92070
Bacillus	*licheniformis*	9789	7750	78	133	53	313900	79960
	pumilus	700814	8200	62250	131	53	108500	81130
	subtilis	6051	4900	3173	151	66	68880	63050
Citrobacter	*braaki*	51113	14450	70	90	48	77660	28430
	freundii	8090	15350	136	138	52	202300	65670
	koseri	25408	28050	56	132	49	44640	36100
Enterococcus	*faecalis*	19433	32700	92	115	49	22490	10540
	faecium	19434	9150	147	132	52	447300	187500
Klebsiella	*oxytoca*	43165	15950	88	175	52	141900	97480
	pneumoniae	4352	56700	46	115	49	22100	249500
Listeria	*innocua*	51742	23100	121	110	49	299100	228900
	ivanovii	19119	82600	88	100	49	368400	110800
	monocytogenes	19115	33150	101	143	71	331400	114900
	welshimeri	35897	9400	156	112	48	253400	181100
Proteus	*mirabilis*	43071	7450	25	117	49	17090	279200
	vulgaris	33420	11600	27	96	49	29170	171800
Shigella	*flexneri*	12022	34500	78	101	52	30360	13950
	sonnei	9290	10900	65	140	48	29360	75300
Streptococcus	*pneumoniae*	6303	34000	75	143	53	178	24610
	pyogenes	12202	1500	142	116	64	193800	91370
Acinetobacter	*baumannii*	19606	16450	78	88	49	232500	113600
Edwardsiella	*tarda*	15947	20200	90	132	52	118200	84540
Enterobacter	*kobei*	BAA-260	11250	118	97	49	455400	180300
Escherichia	*coli*	25922	8850	108	101	71	97980	46360
Hafnia	*alvei*	13337	14850	78	92	49	338500	198800
Moraxella	*catarrhalis*	25238	8350	130	115	53	315800	360300
Morganella	*morganii*	25830	30000	76	212	52	155900	145400
Pluralibacter	*gergoviae*	33028	17400	80	65	49	203400	78990
Pseudomonas	*aeruginosa*	27853	20500	138	142	52	175800	53400
Salmonella	*enterica*	S492	19150	46	121	49	207900	314600
Serratia	*marcescens*	13880	15950	86	91	48	92500	26200
Yersinia	*enterocolitica*	23715	14250	102	112	71	334100	126200
BHI[2]	-	-	-	117	111	49	498000	171000

[1] CFU were determined by plate counting (in duplicate) of either diluted samples for competitors or directly for MRSA. [2] BHI broth was used in place of bacterial culture to identify assay background. [3] For exclusivity, each competitor strain was assessed alone at the indicated CFU per well. [4] For bacterial interference, MRSA (BAA-1720) was added at the burden indicated in combination with the stated competitor CFU per well.

Table S4. CFU and RLU for MRSA screen with clinical *Staphylococcus aureus* (Table 4)

Strain ID	Source	Type	CFU	Control RLU	Selective RLU
BNC 001	Burlington, NC	MRSA	52	479400	39000
BNC 002	Burlington, NC	MRSA	77	407500	305000
BNC 003	Burlington, NC	MRSA	84	588600	143300
BNC 004	Burlington, NC	MRSA	63	183300	210100
BNC 005	Burlington, NC	MRSA	38	358900	22610
BNC 006	Burlington, NC	MRSA	33	18600	24580
BNC 007	Burlington, NC	MRSA	19	190800	3708
BNC 008	Burlington, NC	MRSA	36	86900	1905
BNC 009	Burlington, NC	MRSA	41	522000	109200
BNC 010	Burlington, NC	MRSA	48	363600	1705
BNC 011	Burlington, NC	MRSA	23	609700	412000
BNC 012	Burlington, NC	MRSA	47	1377000	73580
BNC 013	Burlington, NC	MRSA	63	182400	56490
BNC 014	Burlington, NC	MRSA	47	1071000	134500
BNC 015	Burlington, NC	MRSA	22	318700	80540
BNC 016	Burlington, NC	MRSA	40	683200	103900
BNC 017	Burlington, NC	MRSA	44	782800	123000
BNC 018	Burlington, NC	MRSA	36	616200	137200
BNC 019	Burlington, NC	MRSA	52	22610	24300
BNC 020	Burlington, NC	MRSA	80	349000	162200
BNC 021	Burlington, NC	MRSA	52	20110	577
BNC 022	Burlington, NC	MRSA	47	168600	12950
BNC 023	Burlington, NC	MRSA	40	230200	54760
BNC 024	Burlington, NC	MRSA	46	4671	2631
BNC 025	Burlington, NC	MRSA	76	1803000	251700
BNC 026	Burlington, NC	MRSA	100	1185000	246500
BNC 027	Burlington, NC	MRSA	33	1136000	103300
BNC 028	Burlington, NC	MRSA	31	335300	156300
BNC 030	Burlington, NC	MRSA	44	568800	62820
BNC 031	Burlington, NC	MRSA	64	377200	14960
BNC 032	Burlington, NC	MRSA	24	53640	21400
BNC 033	Burlington, NC	MRSA	35	526500	84710
BNC 034	Burlington, NC	MRSA	38	1018000	74260
BNC 035	Burlington, NC	MRSA	42	861600	232900
BNC 036	Burlington, NC	MRSA	41	1136000	3531
BNC 037	Burlington, NC	MRSA	53	694700	229600
BNC 038	Burlington, NC	MRSA	19	106300	70910
BNC 039	Burlington, NC	MRSA	49	101200	34560
BNC 040	Burlington, NC	MRSA	36	152200	34310
BNC 042	Burlington, NC	MRSA	50	690900	155200
BNC 043	Burlington, NC	MRSA	54	576300	49510
BNC 044	Burlington, NC	MRSA	31	748800	26470
BNC 045	Burlington, NC	MRSA	23	329000	50790
BNC 046	Burlington, NC	MRSA	36	52100	26650
BNC 047	Burlington, NC	MRSA	44	375200	71090
BNC 048	Burlington, NC	MRSA	60	656100	200600
BNC 049	Burlington, NC	MRSA	51	138700	9575
BNC 050	Burlington, NC	MRSA	44	326200	41580
BNC 051	Burlington, NC	MRSA	37	423000	141200
BNC 052	Burlington, NC	MRSA	56	713000	80260
BNC 053	Burlington, NC	MRSA	73	1009000	384400
BNC 054	Burlington, NC	MRSA	61	167200	72850
BNC 055	Burlington, NC	MRSA	57	263400	247200
BNC 056	Burlington, NC	MRSA	62	553400	83970
BNC 057	Burlington, NC	MRSA	45	472600	4302
BNC 058	Burlington, NC	MRSA	68	341900	110600
BNC 059	Burlington, NC	MRSA	19	57110	1343

Strain ID	Source	Type	CFU	Control RLU	Selective RLU
BNC 060	Burlington, NC	MRSA	54	517300	63320
BNC 061	Burlington, NC	MRSA	58	844800	254000
BNC 062	Burlington, NC	MRSA	92	120700	31180
BNC 063	Burlington, NC	MRSA	37	158100	342100
BNC 064	Burlington, NC	MRSA	27	68450	276100
BNC 065	Burlington, NC	MRSA	41	266300	56820
BNC 066	Burlington, NC	MRSA	54	142700	14340
BNC 067	Burlington, NC	MRSA	53	220100	343
BNC 068	Burlington, NC	MRSA	28	444500	37440
BNC 069	Burlington, NC	MRSA	20	84730	148000
BNC 070	Burlington, NC	MRSA	44	527700	62200
BNC 071	Burlington, NC	MRSA	20	314400	27510
BNC 072	Burlington, NC	MRSA	16	1711000	69730
BNC 073	Burlington, NC	MRSA	58	753800	395500
BNC 074	Burlington, NC	MRSA	47	749100	277700
BNC 075	Burlington, NC	MRSA	34	487300	379200
BNC 076	Burlington, NC	MRSA	56	1207000	213200
BNC 077	Burlington, NC	MRSA	21	407800	107200
BNC 078	Burlington, NC	MRSA	28	1453000	256600
BNC 079	Burlington, NC	MRSA	37	278600	79120
BNC 080	Burlington, NC	MRSA	30	1149000	278800
BNC 081	Burlington, NC	MRSA	34	739700	360600
BNC 082	Burlington, NC	MRSA	124	253400	346300
BNC 083	Burlington, NC	MRSA	55	335000	26420
BNC 084	Burlington, NC	MRSA	107	1147000	333700
BNC 085	Burlington, NC	MRSA	60	1537000	197300
BNC 086	Burlington, NC	MRSA	72	288600	320800
BNC 087	Burlington, NC	MRSA	67	571900	453700
BNC 088	Burlington, NC	MRSA	63	1197000	459600
BNC 089	Burlington, NC	MRSA	67	610600	210900
BNC 090	Burlington, NC	MRSA	87	615900	245200
BNC 091	Burlington, NC	MRSA	77	1480000	452700
BNC 092	Burlington, NC	MRSA	55	56460	4798
BNC 093	Burlington, NC	MRSA	58	447600	68300
BNC 094	Burlington, NC	MRSA	89	777300	127800
BNC 095	Burlington, NC	MRSA	82	667100	88790
BNC 096	Burlington, NC	MRSA	58	292400	277900
BNC 097	Burlington, NC	MRSA	62	235000	3503
BNC 098	Burlington, NC	MRSA	49	292400	108600
BNC 099	Burlington, NC	MRSA	54	290500	81860
BNC 100	Burlington, NC	MRSA	30	258700	200
BNC 101	Burlington, NC	MRSA	34	9915	201
BNC 102	Burlington, NC	MRSA	99	1417000	619600
BNC 103	Burlington, NC	MRSA	30	960900	129000
BNC 104	Burlington, NC	MRSA	32	24730	5909
BNC 105	Burlington, NC	MRSA	72	65470	10800
BNC 106	Burlington, NC	MRSA	47	461000	31660
BNC 107	Burlington, NC	MRSA	28	1194000	110300
BNC 108	Burlington, NC	MRSA	32	231000	78830
BNC 109	Burlington, NC	MRSA	30	3896	1622
BNC 110	Burlington, NC	MRSA	22	11350	3823
BNC 111	Burlington, NC	MRSA	40	256800	71110
BNC 112	Burlington, NC	MRSA	30	220500	1860
BNC 113	Burlington, NC	MRSA	21	263000	63540
BNC 114	Burlington, NC	MRSA	40	1239000	213000
BNC 115	Burlington, NC	MRSA	88	403400	294800
BNC 116	Burlington, NC	MRSA	119	1482000	539000
BNC 117	Burlington, NC	MRSA	57	733700	882200

Strain ID	Source	Type	CFU	Control RLU	Selective RLU
BNC 118	Burlington, NC	MRSA	40	74430	30210
BNC 119	Burlington, NC	MRSA	77	2284000	33230
BNC 120	Burlington, NC	MRSA	89	1720000	1680000
BNC 121	Burlington, NC	MRSA	71	1905000	1188000
BNC 122	Burlington, NC	MRSA	56	1822000	585800
BNC 123	Burlington, NC	MRSA	99	2689000	1325000
BNC 124	Burlington, NC	MRSA	46	1278000	606200
BNC 125	Burlington, NC	MRSA	66	802100	281100
BNC 126	Burlington, NC	MRSA	49	277200	88590
BNC 127	Burlington, NC	MRSA	41	335900	122000
BNC 128	Burlington, NC	MRSA	60	1709000	348200
BNC 129	Burlington, NC	MRSA	63	1054000	424400
BNC 130	Burlington, NC	MRSA	95	1978000	591800
BNC 131	Burlington, NC	MRSA	48	175600	50620
BNC 132	Burlington, NC	MRSA	43	208000	35030
BNC 133	Burlington, NC	MRSA	36	197100	152800
BNC 134	Burlington, NC	MRSA	62	395200	69230
BNC 135	Burlington, NC	MRSA	60	516200	146000
BNC 136	Burlington, NC	MRSA	23	29540	49420
BNC 137	Burlington, NC	MRSA	28	114500	23060
BNC 138	Burlington, NC	MRSA	46	418900	310100
BNC 139	Burlington, NC	MRSA	42	304700	23260
BNC 140	Burlington, NC	MRSA	32	510500	38020
BNC 141	Burlington, NC	MRSA	48	124000	34990
BNC 142	Burlington, NC	MRSA	152	1351000	195500
BNC 143	Burlington, NC	MRSA	71	1461000	345000
BNC 144	Burlington, NC	MRSA	85	1087000	121300
BNC 145	Burlington, NC	MRSA	66	796300	156000
BNC 146	Burlington, NC	MRSA	77	527500	142700
BNC 147	Burlington, NC	MRSA	46	915500	141400
BNC 148	Burlington, NC	MRSA	9	15650	2590
BNC 149	Burlington, NC	MRSA	70	554300	73420
BNC 150	Burlington, NC	MRSA	88	300300	56170
BNC 151	Burlington, NC	MRSA	41	832900	217300
BNC 152	Burlington, NC	MRSA	53	921300	8959
BNC 153	Burlington, NC	MRSA	32	1144000	254100
BNC 154	Burlington, NC	MRSA	44	700200	680
BNC 155	Burlington, NC	MRSA	59	1015000	82570
BNC 156	Burlington, NC	MRSA	79	560100	298100
BNC 157	Burlington, NC	MRSA	42	523900	458300
BNC 158	Burlington, NC	MRSA	40	974500	78210
BNC 159	Burlington, NC	MRSA	27	116	116
BNC 160	Burlington, NC	MRSA	44	940500	90170
BNC 161	Burlington, NC	MRSA	37	150800	37390
BNC 162	Burlington, NC	MRSA	49	576800	229000
BNC 163	Burlington, NC	MRSA	54	509500	335
BNC 164	Burlington, NC	MRSA	38	126300	9227
BNC 165	Burlington, NC	MRSA	43	220300	73850
BNC 166	Burlington, NC	MRSA	60	52120	11560
BNC 167	Burlington, NC	MRSA	36	943400	136700
BNC 168	Burlington, NC	MRSA	73	1015000	75970
BNC 169	Burlington, NC	MRSA	88	408400	126400
BNC 170	Burlington, NC	MRSA	102	857000	441400
BNC 171	Burlington, NC	MRSA	21	59450	199200
BNC 172	Burlington, NC	MRSA	35	1699000	202000
BNC 173	Burlington, NC	MRSA	59	1823000	4858
BNC 174	Burlington, NC	MRSA	42	1440000	101300
BNC 175	Burlington, NC	MRSA	32	1348000	472100

Strain ID	Source	Type	CFU	Control RLU	Selective RLU
BNC 176	Burlington, NC	MRSA	49	1201000	819400
BNC 177	Burlington, NC	MRSA	31	807900	161300
BNC 178	Burlington, NC	MRSA	45	1473000	125500
BNC 179	Burlington, NC	MRSA	22	231500	319800
BNC 180	Burlington, NC	MRSA	50	1814000	739300
BNC 181	Burlington, NC	MRSA	32	1731000	259400
BNC 182	Burlington, NC	MRSA	50	763000	185900
BNC 183	Burlington, NC	MRSA	37	1103000	96700
BNC 184	Burlington, NC	MRSA	55	706900	321600
BNC 185	Burlington, NC	MRSA	61	2111000	553400
BNC 186	Burlington, NC	MRSA	80	783800	126200
BNC 187	Burlington, NC	MRSA	117	3426000	211500
BNC 188	Burlington, NC	MRSA	72	451100	362100
BNC 189	Burlington, NC	MRSA	45	1372000	149200
BNC 190	Burlington, NC	MRSA	86	3009000	321700
BNC 191	Burlington, NC	MRSA	76	286600	10430
BNC 192	Burlington, NC	MRSA	120	458400	90650
BNC 193	Burlington, NC	MRSA	50	126300	7055
BNC 194	Burlington, NC	MRSA	60	308000	15370
BNC 195	Burlington, NC	MRSA	122	1826000	108100
BNC 196	Burlington, NC	MRSA	68	2787000	888300
BNC 197	Burlington, NC	MRSA	110	1654000	205500
BNC 198	Burlington, NC	MRSA	74	347700	160700
BNC 199	Burlington, NC	MRSA	57	1511000	180700
BNC 200	Burlington, NC	MRSA	66	2263000	110800
BNC 201	Burlington, NC	MRSA	92	1471000	74370
BNC 202	Burlington, NC	MRSA	93	934700	220900
BNC 203	Burlington, NC	MRSA	72	437700	551700
BNC 204	Burlington, NC	MRSA	52	650900	141500
BNC 205	Burlington, NC	MRSA	58	2123000	554600
BNC 206	Burlington, NC	MRSA	72	1709000	61300
BNC 207	Burlington, NC	MRSA	45	1147000	187900
PHX 003	Phoenix, AZ	MRSA	81	801800	359800
PHX 004	Phoenix, AZ	MRSA	87	1038000	803100
PHX 005	Phoenix, AZ	MRSA	112	171200	41110
PHX 006	Phoenix, AZ	MRSA	87	1441000	172600
PHX 007	Phoenix, AZ	MRSA	104	1227000	294200
PHX 008	Phoenix, AZ	MRSA	108	2240000	656800
PHX 009	Phoenix, AZ	MRSA	29	1268000	4035
PHX 010	Phoenix, AZ	MRSA	34	535800	301600
PHX 011	Phoenix, AZ	MRSA	44	794100	74050
PHX 012	Phoenix, AZ	MRSA	27	21470	5567
PHX 013	Phoenix, AZ	MRSA	35	1072000	64470
PHX 014	Phoenix, AZ	MRSA	43	1414000	232200
PHX 015	Phoenix, AZ	MRSA	46	1368000	412800
PHX 016	Phoenix, AZ	MRSA	51	1473000	70650
PHX 017	Phoenix, AZ	MRSA	35	1846000	371400
PHX 019	Phoenix, AZ	MRSA	15	28730	1160
PHX 020	Phoenix, AZ	MRSA	42	1185000	452100
PHX 021	Phoenix, AZ	MRSA	75	310200	161100
PHX 022	Phoenix, AZ	MRSA	52	2445000	769600
PHX 023	Phoenix, AZ	MRSA	72	371400	198900
PHX 024	Phoenix, AZ	MRSA	45	1120000	166400
PHX 025	Phoenix, AZ	MRSA	16	120500	61620
PHX 026	Phoenix, AZ	MRSA	7	1076000	120200
PHX 027	Phoenix, AZ	MRSA	68	1282000	428100
PHX 028	Phoenix, AZ	MRSA	66	1072000	425000
PHX 029	Phoenix, AZ	MRSA	47	966500	238100

Strain ID	Source	Type	CFU	Control RLU	Selective RLU
PHX 030	Phoenix, AZ	MRSA	53	109300	8256
PHX 031	Phoenix, AZ	MRSA	25	29020	1695
PHX 032	Phoenix, AZ	MRSA	52	1163000	361900
PHX 033	Phoenix, AZ	MRSA	20	1807000	364600
PHX 034	Phoenix, AZ	MRSA	41	1075000	255100
PHX 035	Phoenix, AZ	MRSA	29	827200	209100
PHX 036	Phoenix, AZ	MRSA	29	95790	32800
PHX 037	Phoenix, AZ	MRSA	45	184500	34990
PHX 038	Phoenix, AZ	MRSA	58	212500	96450
PHX 039	Phoenix, AZ	MRSA	64	360800	50240
PHX 040	Phoenix, AZ	MRSA	28	1852000	819200
PHX 041	Phoenix, AZ	MRSA	21	202400	6988
PHX 042	Phoenix, AZ	MRSA	28	16570	860
PHX 043	Phoenix, AZ	MRSA	41	1824000	716300
PHX 044	Phoenix, AZ	MRSA	79	431300	79640
PHX 045	Phoenix, AZ	MRSA	130	525900	148000
PHX 046	Phoenix, AZ	MRSA	81	335800	61970
PHX 047	Phoenix, AZ	MRSA	44	1420000	246700
PHX 048	Phoenix, AZ	MRSA	39	143800	3399
PHX 049	Phoenix, AZ	MRSA	40	1116000	147400
PHX 050	Phoenix, AZ	MRSA	115	688400	130800
PHX 051	Phoenix, AZ	MRSA	46	2213000	406000
PHX 052	Phoenix, AZ	MRSA	67	8380	9214
PHX 053	Phoenix, AZ	MRSA	87	824000	782
PHX 054	Phoenix, AZ	MRSA	143	2480000	407800
PHX 055	Phoenix, AZ	MRSA	82	2912000	1214000
PHX 056	Phoenix, AZ	MRSA	53	306800	167500
PHX 057	Phoenix, AZ	MRSA	44	1611000	430900
PHX 058	Phoenix, AZ	MRSA	62	1386000	221300
PHX 059	Phoenix, AZ	MRSA	101	2572000	949300
PHX 060	Phoenix, AZ	MRSA	53	1594000	622800
PHX 061	Phoenix, AZ	MRSA	65	108900	80590
PHX 062	Phoenix, AZ	MRSA	16	1534000	24560
PHX 063	Phoenix, AZ	MRSA	24	1041000	23500
PHX 064	Phoenix, AZ	MRSA	19	1346000	67730
PHX 065	Phoenix, AZ	MRSA	37	1076000	58500
PHX 066	Phoenix, AZ	MRSA	41	1381000	171400
PHX 067	Phoenix, AZ	MRSA	37	1501000	323600
PHX 068	Phoenix, AZ	MRSA	35	342600	132900
PHX 069	Phoenix, AZ	MRSA	106	1188000	155600
PHX 070	Phoenix, AZ	MRSA	75	1182000	111500
PHX 071	Phoenix, AZ	MRSA	84	140200	30350
PHX 072	Phoenix, AZ	MRSA	9	86350	3319
PHX 073	Phoenix, AZ	MRSA	31	224500	2761
PHX 074	Phoenix, AZ	MRSA	51	1434000	510000
PHX 075	Phoenix, AZ	MRSA	32	1474000	239800
PHX 076	Phoenix, AZ	MRSA	58	544900	146400
PHX 077	Phoenix, AZ	MRSA	49	295200	5176
PHX 079	Phoenix, AZ	MRSA	58	582	251
PHX 080	Phoenix, AZ	MRSA	44	276500	756
PHX 081	Phoenix, AZ	MRSA	60	747000	92300
PHX 082	Phoenix, AZ	MRSA	18	415900	50380
PHX 083	Phoenix, AZ	MRSA	61	769500	477100
PHX 084	Phoenix, AZ	MRSA	57	482800	173700
PHX 085	Phoenix, AZ	MRSA	21	819100	988
PHX 086	Phoenix, AZ	MRSA	20	16720	4286
PHX 087	Phoenix, AZ	MRSA	40	491100	34790
PHX 088	Phoenix, AZ	MRSA	28	986100	611400

Strain ID	Source	Type	CFU	Control RLU	Selective RLU
PHX 089	Phoenix, AZ	MRSA	17	923000	381100
PHX 090	Phoenix, AZ	MRSA	32	1217000	75950
PHX 091	Phoenix, AZ	MRSA	36	22040	1321
PHX 092	Phoenix, AZ	MRSA	58	1093000	256800
PHX 093	Phoenix, AZ	MRSA	61	1687000	565000
PHX 094	Phoenix, AZ	MRSA	41	1611000	459200
PHX 095	Phoenix, AZ	MRSA	1	14410	4688
PHX 096	Phoenix, AZ	MRSA	32	481300	445900
PHX 097	Phoenix, AZ	MRSA	55	1311000	70120
PHX 098	Phoenix, AZ	MRSA	54	400400	241900
PHX 099	Phoenix, AZ	MRSA	27	247600	26080
PHX 100	Phoenix, AZ	MRSA	54	600100	12280
RNJ 002	Raritan, NJ	MRSA	62	29810	16180
RNJ 003	Raritan, NJ	MRSA	35	3892	3572
RNJ 004	Raritan, NJ	MRSA	52	642500	66700
RNJ 005	Raritan, NJ	MRSA	52	31490	615
RNJ 006	Raritan, NJ	MRSA	59	44020	1850
RNJ 007	Raritan, NJ	MRSA	38	354200	138600
RNJ 008	Raritan, NJ	MRSA	24	254700	18220
RNJ 009	Raritan, NJ	MRSA	29	23730	4994
RNJ 010	Raritan, NJ	MRSA	40	84270	13800
RNJ 011	Raritan, NJ	MRSA	49	930900	276600
RNJ 013	Raritan, NJ	MRSA	43	437600	85070
RNJ 014	Raritan, NJ	MRSA	63	214500	89000
RNJ 015	Raritan, NJ	MRSA	58	160700	7251
RNJ 016	Raritan, NJ	MRSA	30	213400	48850
RNJ 017	Raritan, NJ	MRSA	61	1040000	121600
RNJ 019	Raritan, NJ	MRSA	54	1665	555
RNJ 020	Raritan, NJ	MRSA	25	353100	6338
RNJ 021	Raritan, NJ	MRSA	26	163100	31140
RNJ 022	Raritan, NJ	MRSA	44	267800	61040
RNJ 023	Raritan, NJ	MRSA	30	1439000	117700
RNJ 024	Raritan, NJ	MRSA	17	434000	2368
RNJ 025	Raritan, NJ	MRSA	76	31360	1928
RNJ 026	Raritan, NJ	MRSA	34	779200	21940
RNJ 027	Raritan, NJ	MRSA	23	1209000	115500
RNJ 028	Raritan, NJ	MRSA	51	1016000	255000
RNJ 029	Raritan, NJ	MRSA	74	221900	54640
RNJ 030	Raritan, NJ	MRSA	28	390600	126900
RNJ 031	Raritan, NJ	MRSA	59	230900	105700
RNJ 033	Raritan, NJ	MRSA	44	768600	395500
RNJ 034	Raritan, NJ	MRSA	83	560700	61870
RNJ 035	Raritan, NJ	MRSA	64	1620000	320000
RNJ 036	Raritan, NJ	MRSA	59	752200	157100
RNJ 037	Raritan, NJ	MRSA	5	583000	111500
RNJ 038	Raritan, NJ	MRSA	81	1206000	182100
RNJ 039	Raritan, NJ	MRSA	33	1107000	4091
RNJ 040	Raritan, NJ	MRSA	59	6722	2635
RNJ 041	Raritan, NJ	MRSA	39	651100	466
RNJ 042	Raritan, NJ	MRSA	34	375500	64700
RNJ 043	Raritan, NJ	MRSA	31	1331000	160000
RNJ 044	Raritan, NJ	MRSA	45	1941000	298400
RNJ 045	Raritan, NJ	MRSA	12	1499000	293600
RNJ 046	Raritan, NJ	MRSA	53	695600	266500
RNJ 047	Raritan, NJ	MRSA	40	938000	168200
RNJ 048	Raritan, NJ	MRSA	67	1339000	265200
RNJ 049	Raritan, NJ	MRSA	33	376500	132000
RNJ 050	Raritan, NJ	MRSA	17	325100	2277

Strain ID	Source	Type	CFU	Control RLU	Selective RLU
RNJ 051	Raritan, NJ	MRSA	48	791500	667500
RNJ 052	Raritan, NJ	MRSA	58	659900	375000
RNJ 053	Raritan, NJ	MRSA	55	2820000	759100
RNJ 054	Raritan, NJ	MRSA	53	415200	70710
RNJ 055	Raritan, NJ	MRSA	26	822600	167700
RNJ 056	Raritan, NJ	MRSA	65	1421000	60460
RNJ 057	Raritan, NJ	MRSA	34	1070000	251600
RNJ 058	Raritan, NJ	MRSA	34	1700000	326400
RNJ 063	Raritan, NJ	MRSA	45	742200	173000
RNJ 064	Raritan, NJ	MRSA	26	431600	91070
RNJ 067	Raritan, NJ	MRSA	40	1264000	326800
RNJ 068	Raritan, NJ	MRSA	31	1492000	976600
RNJ 069	Raritan, NJ	MRSA	24	500000	356000
RNJ 070	Raritan, NJ	MRSA	43	1720000	197700
RNJ 072	Raritan, NJ	MRSA	29	389700	172700
RNJ 073	Raritan, NJ	MRSA	25	391600	103700
RNJ 074	Raritan, NJ	MRSA	56	1484000	522600
RNJ 075	Raritan, NJ	MRSA	48	1287000	46680
RNJ 076	Raritan, NJ	MRSA	55	3062000	41000
RNJ 077	Raritan, NJ	MRSA	51	457500	501100
RNJ 078	Raritan, NJ	MRSA	56	1717000	702100
RNJ 079	Raritan, NJ	MRSA	63	396300	116600
RNJ 080	Raritan, NJ	MRSA	45	889400	291400
RNJ 081	Raritan, NJ	MRSA	45	214200	85150
RNJ 082	Raritan, NJ	MRSA	84	418900	100300
RNJ 083	Raritan, NJ	MRSA	61	9127	1862
RNJ 084	Raritan, NJ	MRSA	27	42700	7484
RNJ 085	Raritan, NJ	MRSA	32	66280	7761
RNJ 086	Raritan, NJ	MRSA	37	849200	98460
RNJ 087	Raritan, NJ	MRSA	75	277800	104300
RNJ 088	Raritan, NJ	MRSA	24	390600	40130
RNJ 089	Raritan, NJ	MRSA	26	757500	81980
RNJ 090	Raritan, NJ	MRSA	45	1142000	99340
RNJ 091	Raritan, NJ	MRSA	33	457500	38900
RNJ 092	Raritan, NJ	MRSA	51	720300	81470
RNJ 093	Raritan, NJ	MRSA	15	92420	23290
RNJ 094	Raritan, NJ	MRSA	20	615000	20570
RNJ 095	Raritan, NJ	MRSA	116	1033000	53080
RNJ 096	Raritan, NJ	MRSA	53	475800	22780
RNJ 097	Raritan, NJ	MRSA	53	1277000	105100
RNJ 098	Raritan, NJ	MRSA	54	430900	32150
RNJ 099	Raritan, NJ	MRSA	64	534900	195100
RNJ 100	Raritan, NJ	MRSA	48	1680000	398200
MSSA 001	Burlington, NC	MSSA	1115	60090000	142
MSSA 001	Burlington, NC	MSSA	11150	191600000	151
MSSA 002	Burlington, NC	MSSA	1030	22240000	142
MSSA 002	Burlington, NC	MSSA	10300	103900000	302
MSSA 003	Burlington, NC	MSSA	555	1107000	152
MSSA 003	Burlington, NC	MSSA	5550	588700	256
MSSA 004	Burlington, NC	MSSA	520	505800	172
MSSA 004	Burlington, NC	MSSA	5200	658000	167
MSSA 005	Burlington, NC	MSSA	760	26650000	156
MSSA 005	Burlington, NC	MSSA	7600	218500000	826
MSSA 006	Burlington, NC	MSSA	850	17170000	271
MSSA 006	Burlington, NC	MSSA	8500	102100000	1296
MSSA 007	Burlington, NC	MSSA	890	30170000	130
MSSA 007	Burlington, NC	MSSA	8900	173300000	192
MSSA 008	Burlington, NC	MSSA	495	32320000	130

Strain ID	Source	Type	CFU	Control RLU	Selective RLU
MSSA 008	Burlington, NC	MSSA	4950	178400000	355
MSSA 009	Burlington, NC	MSSA	975	10060000	131
MSSA 009	Burlington, NC	MSSA	9750	105400000	133
MSSA 010	Burlington, NC	MSSA	530	584200	143
MSSA 010	Burlington, NC	MSSA	5300	16930000	145
MSSA 012	Burlington, NC	MSSA	1505	844200	145
MSSA 012	Burlington, NC	MSSA	15050	228400	152
MSSA 013	Burlington, NC	MSSA	685	8570000	151
MSSA 013	Burlington, NC	MSSA	6850	69550000	130
MSSA 014	Burlington, NC	MSSA	725	5493000	211
MSSA 014	Burlington, NC	MSSA	7250	32060000	783
MSSA 015	Burlington, NC	MSSA	835	5210000	160
MSSA 015	Burlington, NC	MSSA	8350	61260000	153
MSSA 016	Burlington, NC	MSSA	640	4549000	153
MSSA 016	Burlington, NC	MSSA	6400	69860000	187
MSSA 017	Burlington, NC	MSSA	615	11440000	160
MSSA 017	Burlington, NC	MSSA	6150	80940000	140
MSSA 018	Burlington, NC	MSSA	800	8989000	133
MSSA 018	Burlington, NC	MSSA	8000	86910000	210
MSSA 019	Burlington, NC	MSSA	750	5678000	155
MSSA 019	Burlington, NC	MSSA	7500	47380000	221
MSSA 020	Burlington, NC	MSSA	770	5347000	171
MSSA 020	Burlington, NC	MSSA	7700	60860000	568
MSSA 021	Burlington, NC	MSSA	820	5190000	142
MSSA 021	Burlington, NC	MSSA	8200	51500000	180
MSSA 022	Burlington, NC	MSSA	515	4629000	147
MSSA 022	Burlington, NC	MSSA	5150	40440000	115
MSSA 023	Burlington, NC	MSSA	1190	3793000	152
MSSA 023	Burlington, NC	MSSA	11900	37420000	201
MSSA 024	Burlington, NC	MSSA	840	10200000	186
MSSA 024	Burlington, NC	MSSA	8400	71780000	228
MSSA 025	Burlington, NC	MSSA	5840	50060000	143
MSSA 025	Burlington, NC	MSSA	58400	9237000	257
MSSA 026	Burlington, NC	MSSA	967	15230000	121
MSSA 026	Burlington, NC	MSSA	9669	143300000	2617
MSSA 027	Burlington, NC	MSSA	574	5188000	111
MSSA 027	Burlington, NC	MSSA	5739	24030000	135
MSSA 028	Burlington, NC	MSSA	392	30070000	166
MSSA 028	Burlington, NC	MSSA	3918	116000000	3060
MSSA 029	Burlington, NC	MSSA	815	26130000	205
MSSA 029	Burlington, NC	MSSA	8146	186600000	2990
MSSA 030	Burlington, NC	MSSA	602	7841000	150
MSSA 030	Burlington, NC	MSSA	6021	20930000	115
MSSA 031	Burlington, NC	MSSA	585	65420000	126
MSSA 031	Burlington, NC	MSSA	5850	173000000	221
MSSA 032	Burlington, NC	MSSA	691	482400	118
MSSA 032	Burlington, NC	MSSA	6905	692000	112
MSSA 033	Burlington, NC	MSSA	75	32220000	51460
MSSA 033	Burlington, NC	MSSA	750	2769000	991900
MSSA 034	Burlington, NC	MSSA	665	81260	1785
MSSA 034	Burlington, NC	MSSA	6650	662100	22640
MSSA 035	Burlington, NC	MSSA	890	9391000	110
MSSA 035	Burlington, NC	MSSA	8900	21660000	123
MSSA 036	Burlington, NC	MSSA	650	14430000	110
MSSA 036	Burlington, NC	MSSA	6500	116500000	130
MSSA 037	Burlington, NC	MSSA	1310	99810000	155
MSSA 037	Burlington, NC	MSSA	13100	139200000	127
MSSA 038	Burlington, NC	MSSA	2650	19110000	135

Strain ID	Source	Type	CFU	Control RLU	Selective RLU
MSSA 038	Burlington, NC	MSSA	26500	4454000	138
MSSA 040	Burlington, NC	MSSA	630	19320000	142
MSSA 040	Burlington, NC	MSSA	6300	130600000	262
MSSA 041	Burlington, NC	MSSA	575	6440000	136
MSSA 041	Burlington, NC	MSSA	5750	11370000	127
MSSA 042	Burlington, NC	MSSA	1065	17930000	93
MSSA 042	Burlington, NC	MSSA	10650	148500000	123
MSSA 043	Burlington, NC	MSSA	1135	11780	105
MSSA 043	Burlington, NC	MSSA	11350	9546	138
MSSA 044	Burlington, NC	MSSA	675	35030000	126
MSSA 044	Burlington, NC	MSSA	6750	22690000	125
MSSA 045	Burlington, NC	MSSA	380	117500000	138
MSSA 045	Burlington, NC	MSSA	3800	127500000	198
MSSA 046	Burlington, NC	MSSA	1820	21270000	6603
MSSA 046	Burlington, NC	MSSA	18200	83650000	51970
MSSA 047	Burlington, NC	MSSA	525	12970000	101
MSSA 047	Burlington, NC	MSSA	5250	112400000	130
MSSA 048	Burlington, NC	MSSA	605	80400	127
MSSA 048	Burlington, NC	MSSA	6050	112400	142
MSSA 049	Burlington, NC	MSSA	1155	9999000	111
MSSA 049	Burlington, NC	MSSA	11550	94090000	127
MSSA 051	Burlington, NC	MSSA	1070	49090000	102
MSSA 051	Burlington, NC	MSSA	10698	166400000	230
MSSA 052	Burlington, NC	MSSA	1027	58950	118
MSSA 052	Burlington, NC	MSSA	10273	63240	6854
MSSA 053	Burlington, NC	MSSA	2470	13200000	136
MSSA 053	Burlington, NC	MSSA	24700	1841000	116
MSSA 054	Burlington, NC	MSSA	1163	21280000	141
MSSA 054	Burlington, NC	MSSA	11626	95230000	242
MSSA 055	Burlington, NC	MSSA	524	32020	122
MSSA 055	Burlington, NC	MSSA	5244	397100	143
MSSA 056	Burlington, NC	MSSA	1645	167500	115
MSSA 056	Burlington, NC	MSSA	16445	139800	216
MSSA 057	Burlington, NC	MSSA	4010	354000	140
MSSA 057	Burlington, NC	MSSA	40100	89330	127
MSSA 058	Burlington, NC	MSSA	834	23450000	191
MSSA 058	Burlington, NC	MSSA	8337	164100000	403
MSSA 059	Burlington, NC	MSSA	620	4902000	146
MSSA 059	Burlington, NC	MSSA	6200	3845000	195
MSSA 060	Burlington, NC	MSSA	600	28390000	137
MSSA 060	Burlington, NC	MSSA	6000	236600000	131
MSSA 062	Burlington, NC	MSSA	590	29090000	173
MSSA 062	Burlington, NC	MSSA	5900	186300000	145
MSSA 063	Burlington, NC	MSSA	835	32080000	151
MSSA 063	Burlington, NC	MSSA	8350	16880000	227
MSSA 064	Burlington, NC	MSSA	730	11270000	117
MSSA 064	Burlington, NC	MSSA	7300	96400000	152
MSSA 065	Burlington, NC	MSSA	850	4029000	125
MSSA 065	Burlington, NC	MSSA	8500	32560000	223
MSSA 066	Burlington, NC	MSSA	915	26320000	123
MSSA 066	Burlington, NC	MSSA	9150	182100000	208
MSSA 067	Burlington, NC	MSSA	895	509900	125
MSSA 067	Burlington, NC	MSSA	8950	224900	122
MSSA 068	Burlington, NC	MSSA	1215	22720000	647
MSSA 068	Burlington, NC	MSSA	12150	123100000	6137
MSSA 070	Burlington, NC	MSSA	850	2636000	140
MSSA 070	Burlington, NC	MSSA	8500	2484000	243
MSSA 071	Burlington, NC	MSSA	940	32020000	101

Strain ID	Source	Type	CFU	Control RLU	Selective RLU
MSSA 071	Burlington, NC	MSSA	9400	200800000	153
MSSA 072	Burlington, NC	MSSA	555	12640000	132
MSSA 072	Burlington, NC	MSSA	5550	35260000	191
MSSA 073	Burlington, NC	MSSA	1240	146000000	257
MSSA 073	Burlington, NC	MSSA	12400	153900000	986
MSSA 074	Burlington, NC	MSSA	795	38360000	127
MSSA 074	Burlington, NC	MSSA	7950	204600000	241
MSSA 075	Burlington, NC	MSSA	505	2452000	135
MSSA 075	Burlington, NC	MSSA	5050	4719000	155
MSSA 076	Burlington, NC	MSSA	560	26460000	155
MSSA 076	Burlington, NC	MSSA	5600	19290000	143
MSSA 077	Burlington, NC	MSSA	612	13580	136
MSSA 077	Burlington, NC	MSSA	6117	114600	146
MSSA 078	Burlington, NC	MSSA	600	12790000	237
MSSA 078	Burlington, NC	MSSA	5996	111100000	176
MSSA 079	Burlington, NC	MSSA	845	3778000	1992
MSSA 079	Burlington, NC	MSSA	8450	468000	69570
MSSA 081	Burlington, NC	MSSA	508	3471000	143
MSSA 081	Burlington, NC	MSSA	5082	10920000	178
MSSA 082	Burlington, NC	MSSA	1190	752100	146
MSSA 082	Burlington, NC	MSSA	11903	1879000	2578
MSSA 083	Burlington, NC	MSSA	1241	8341000	157
MSSA 083	Burlington, NC	MSSA	12413	7199000	251
MSSA 084	Burlington, NC	MSSA	653	19390000	127
MSSA 084	Burlington, NC	MSSA	6533	220200000	19330
MSSA 085	Burlington, NC	MSSA	1795	99440000	150
MSSA 085	Burlington, NC	MSSA	17950	146200000	163
MSSA 086	Burlington, NC	MSSA	520	49250000	2293
MSSA 086	Burlington, NC	MSSA	5200	105600000	493
MSSA 087	Burlington, NC	MSSA	1775	3066000	146
MSSA 087	Burlington, NC	MSSA	17750	685800	121
MSSA 088	Burlington, NC	MSSA	560	30040000	146
MSSA 088	Burlington, NC	MSSA	5600	206200000	150
MSSA 089	Burlington, NC	MSSA	530	36470000	127
MSSA 089	Burlington, NC	MSSA	5300	42370000	146
MSSA 090	Burlington, NC	MSSA	2665	410	136
MSSA 090	Burlington, NC	MSSA	26650	362	132
MSSA 092	Burlington, NC	MSSA	1720	12890000	242
MSSA 092	Burlington, NC	MSSA	17200	9981000	151
MSSA 093	Burlington, NC	MSSA	815	13200000	146
MSSA 093	Burlington, NC	MSSA	8150	139500000	147
MSSA 094	Burlington, NC	MSSA	540	30770000	308
MSSA 094	Burlington, NC	MSSA	5400	217700000	2002
MSSA 095	Burlington, NC	MSSA	550	3105000	148
MSSA 095	Burlington, NC	MSSA	5500	6685000	163
MSSA 096	Burlington, NC	MSSA	520	4198000	137
MSSA 096	Burlington, NC	MSSA	5200	1380000	223
MSSA 097	Burlington, NC	MSSA	1075	38180000	141
MSSA 097	Burlington, NC	MSSA	10750	173600000	145
MSSA 098	Burlington, NC	MSSA	620	92970000	160
MSSA 098	Burlington, NC	MSSA	6200	112700000	192
MSSA 099	Burlington, NC	MSSA	500	25140000	138
MSSA 099	Burlington, NC	MSSA	5000	14820000	133
MSSA 100	Burlington, NC	MSSA	2465	665900	161
MSSA 100	Burlington, NC	MSSA	24650	261600	131
MSSA 101	Burlington, NC	MSSA	1775	17240000	640
MSSA 101	Burlington, NC	MSSA	17750	100700000	6379
MSSA 102	Burlington, NC	MSSA	1305	57270000	116

Strain ID	Source	Type	CFU	Control RLU	Selective RLU
MSSA 102	Burlington, NC	MSSA	13050	123300000	196
MSSA 103	Burlington, NC	MSSA	1310	659300	111
MSSA 103	Burlington, NC	MSSA	13100	1076000	202
MSSA 104	Burlington, NC	MSSA	865	122100000	163
MSSA 104	Burlington, NC	MSSA	8650	196100000	230
MSSA 105	Burlington, NC	MSSA	605	233100	260
MSSA 105	Burlington, NC	MSSA	6050	3094000	401
MSSA 106	Burlington, NC	MSSA	2170	294600	138
MSSA 106	Burlington, NC	MSSA	21700	1219000	233
MSSA 107	Burlington, NC	MSSA	2170	58870000	131
MSSA 107	Burlington, NC	MSSA	21700	178100000	187
MSSA 108	Burlington, NC	MSSA	1470	34780000	121
MSSA 108	Burlington, NC	MSSA	14700	16800000	150
MSSA 109	Burlington, NC	MSSA	1075	211400	127
MSSA 109	Burlington, NC	MSSA	10750	193200	141
MSSA 110	Burlington, NC	MSSA	1940	105800000	141
MSSA 110	Burlington, NC	MSSA	19400	157500000	148
MSSA 111	Burlington, NC	MSSA	1295	64950000	142
MSSA 111	Burlington, NC	MSSA	12950	163300000	150
MSSA 112	Burlington, NC	MSSA	1715	69600000	142
MSSA 112	Burlington, NC	MSSA	17150	96770000	447
MSSA 114	Burlington, NC	MSSA	2650	29930000	167
MSSA 114	Burlington, NC	MSSA	26500	80920000	276
MSSA 115	Burlington, NC	MSSA	1240	7031000	3608
MSSA 115	Burlington, NC	MSSA	12400	1038000	22410
MSSA 116	Burlington, NC	MSSA	660	14330000	132
MSSA 116	Burlington, NC	MSSA	6600	7542000	838
MSSA 117	Burlington, NC	MSSA	1575	32730000	117
MSSA 117	Burlington, NC	MSSA	15750	17000000	281
MSSA 118	Burlington, NC	MSSA	370	31230000	127
MSSA 118	Burlington, NC	MSSA	3700	96050000	140
MSSA 119	Burlington, NC	MSSA	1260	2231000	180
MSSA 119	Burlington, NC	MSSA	12600	31030000	536
MSSA 120	Burlington, NC	MSSA	1690	1872000	132
MSSA 120	Burlington, NC	MSSA	16900	78730000	120
MSSA 121	Burlington, NC	MSSA	1010	105100000	187
MSSA 121	Burlington, NC	MSSA	10100	164900000	940
MSSA 122	Burlington, NC	MSSA	755	57600000	181
MSSA 122	Burlington, NC	MSSA	7550	29350000	553
MSSA 123	Burlington, NC	MSSA	910	68570000	138
MSSA 123	Burlington, NC	MSSA	9100	253500000	147
MSSA 124	Burlington, NC	MSSA	1695	79390000	153
MSSA 124	Burlington, NC	MSSA	16950	137800000	135
MSSA 127	Burlington, NC	MSSA	2705	26150000	572
MSSA 127	Burlington, NC	MSSA	27050	9820000	547
MSSA 128	Burlington, NC	MSSA	950	39710000	245
MSSA 128	Burlington, NC	MSSA	9500	175000000	1935
MSSA 129	Burlington, NC	MSSA	1315	16500000	153
MSSA 129	Burlington, NC	MSSA	13150	23170000	157
MSSA 130	Burlington, NC	MSSA	1465	43860000	105
MSSA 130	Burlington, NC	MSSA	14650	148700000	153
MSSA 131	Burlington, NC	MSSA	1250	39310000	140
MSSA 131	Burlington, NC	MSSA	12500	24780000	153
MSSA 132	Burlington, NC	MSSA	1545	54710000	167
MSSA 132	Burlington, NC	MSSA	15450	27560000	338
MSSA 133	Burlington, NC	MSSA	1245	51020000	172
MSSA 133	Burlington, NC	MSSA	12450	188000000	206

Table S5. CFU and RLU for nasal swabs: endogenous, MRSA spike, and autoluminescence (Table 5)

	RLU for Endogenous[1] (Elutant only)		RLU for MRSA Spike[2] (Elutant + MRSA)				RLU for Autoluminescence[3] (No luciferase)
**Swab #**	**Control**	**Selective**	**Strain**	**CFU[4]**	**Control**	**Selective**	**-**
1	745100	451	BAA-1707	65	4932000	199400	331
2	161	163	BAA-1707	65	3183000	1955000	22
3	227	193	BAA-1707	65	2070000	339500	72
4	4778	197	BAA-1707	65	3371000	1353000	41
5	19310	215	BAA-1707	65	4343000	1564000	30
6	8334	240	BAA-1707	65	4523000	991800	33
7	7619	195	BAA-1707	65	3153000	1167000	47
8	34800	171	BAA-1707	65	4569000	1178000	30
9	54630	225	BAA-1717	105	2226000	167600	115
10	28380	198	BAA-1717	105	4176000	756500	33
11	56130	1081	BAA-1717	105	2877000	267300	58
12	6182	200	BAA-1717	105	4740000	467100	62
13	1536000	6505	BAA-1717	105	1714000	196300	153
14	27190	150	BAA-1717	105	2649000	288100	23
15	8236	157	BAA-1717	105	3584000	423200	23
16	680	197	BAA-1717	105	463500	41230	86
17	1096	178	BAA-1720	111	1651000	401600	70
18	89100	1437	BAA-1720	111	801100	60590	90
19	2880	158	BAA-1720	111	1076000	257300	38
20	48020	195	BAA-1720	111	622800	59180	65
21	1280	132	BAA-1720	111	805600	163400	38
22	1446	192	BAA-1720	111	317000	5885	82
23	46610	165	BAA-1720	111	1085000	201700	52
24	685	140	BAA-1720	111	835600	209800	28
25	78950	152	BAA-1763	87	520600	45140	41
26	136400	160	BAA-1763	87	296100	34190	47
27	3588	177	BAA-1763	87	804900	56840	32
28	4231	170	BAA-1763	87	653000	36440	38
29	104200	157	BAA-1763	87	526900	59530	45
30	662	198	BAA-1763	87	250200	25280	37
31	33430000	390	BAA-1763	87	41320000	3571	270
32	848600	253	BAA-1763	87	624500	20680	141
33	145700	4303	BAA-1766	79	569900	880600	205
34	81410	265	BAA-1766	79	4799000	583300	190
35	16140	202	BAA-1766	79	3203000	823	81
36	36630	202	BAA-1766	79	5204000	539800	81
37	372	126	BAA-1766	79	3717000	436100	27
38	2223	150	BAA-1766	79	3976000	369900	18
39	1160	190	BAA-1766	79	3502000	511100	53
40	341	171	BAA-1766	79	1688000	85880	75
BHI	-	-	BAA-1707	65	2112000	900500	-
BHI	-	-	BAA-1717	105	614300	43590	-
BHI	-	-	BAA-1720	111	245100	147900	-
BHI	-	-	BAA-1763	87	35380	6080	-
BHI	-	-	BAA-1766	79	191300	10460	-
BHI	126	85	-	-	-	-	22

[1] Nasal swabs were eluted in BHI and assayed directly. [2] Nasal elutants were spiked with the indicated MRSA strain at the stated CFU per well. [3] Nasal elutants were combined with luciferase substrate and buffer in the absence of luciferase reporter phage. Signal in these wells is considered to be autoluminescence, likely the result of non-specific activation of the substrate or pre-existing luminescence in the sample. [4] CFU per well were determined directly by plate counting (in duplicate).
